# Improving eukaryotic genome annotation using single molecule mRNA sequencing

**DOI:** 10.1186/s12864-018-4555-7

**Published:** 2018-03-01

**Authors:** Vincent Magrini, Xin Gao, Bruce A. Rosa, Sean McGrath, Xu Zhang, Kymberlie Hallsworth-Pepin, John Martin, John Hawdon, Richard K. Wilson, Makedonka Mitreva

**Affiliations:** 1 McDonnell Genome Institute, Washington University School of Medicine, St. Louis, MO 63108 USA; 2 Department of Microbiology, Immunology and Tropical Medicine, The George Washington University, Washington DC, 20037 USA; 3 Department of Medicine, Washington University School of Medicine, St. Louis, MO 63110 USA

**Keywords:** Genome annotation improvement, Pacific bioscience mRNA sequencing, *Ancylostoma ceylanicum*, Hookworm, Gene loci

## Abstract

**Background:**

The advantages of Pacific Biosciences (PacBio) single-molecule real-time (SMRT) technology include long reads, low systematic bias, and high consensus read accuracy. Here we use these attributes to improve on the genome annotation of the parasitic hookworm *Ancylostoma ceylanicum* using PacBio RNA-Seq*.*

**Results:**

We sequenced 192,888 circular consensus sequences (CCS) derived from cDNAs generated using the CloneTech SMARTer system. These SMARTer-SMRT libraries were normalized and size-selected providing a robust population of expressed structural genes for subsequent genome annotation. We demonstrate PacBio mRNA sequences based genome annotation improvement, compared to genome annotation using conventional sequencing-by-synthesis alone, by identifying 1609 (9.2%) new genes, extended the length of 3965 (26.7%) genes and increased the total genomic exon length by 1.9 Mb (12.4%). Non-coding sequence representation (primarily from UTRs based on dT reverse transcription priming) was particularly improved, increasing in total length by fifteen-fold, by increasing both the length and number of UTR exons. In addition, the UTR data provided by these CCS allowed for the identification of a novel SL2 splice leader sequence for *A. ceylanicum* and an increase in the number and proportion of functionally annotated genes. RNA-seq data also confirmed some of the newly annotated genes and gene features.

**Conclusion:**

Overall, PacBio data has supported a significant improvement in gene annotation in this genome, and is an appealing alternative or complementary technique for genome annotation to the other transcript sequencing technologies.

**Electronic supplementary material:**

The online version of this article (10.1186/s12864-018-4555-7) contains supplementary material, which is available to authorized users.

## Background

Compared to conventional 454/Roche and/or Illumina sequencing platforms, the Pacific Biosciences’s (PacBio) much longer reads and improved accuracy using circular consensus sequences (CCS) are advantageous for sequencing cDNA libraries because i) each library read is from a single transcript molecule, ii) mRNA CCS lengths on PacBio easily exceed 1kbp, iii) the longer reads provide a unique opportunity to identify 5′ and 3′ boundaries or untranslated regions (UTRs), and iv) for each gene, multiple transcripts may exist and long read sequencing provides exon-exon boundaries that discriminate isoforms and novel fusions. In contrast to short read technologies, the disadvantage of obtaining a reduced dynamic range of gene expression is irrelevant when the goal is accurate gene annotation.

To understand genome organization and genic content, whole genome shotgun approaches have been traditionally assembled from short read NGS technologies such as the 454/Roche and/or Illumina platform for many organisms including nematodes [1–3]. The quality of the published draft assemblies is variable (Table 1; e.g. [1, 3]; Accession numbers of all published nematode genomes are available on Nematode.net) and may result in suboptimal downstream comparative genomic analysis including gene annotation. Thus, examining approaches that use other technologies (such as the long read single-molecule mRNA sequencing) to improve annotation of already existing genome assemblies in GenBank is needed.

**Table 1:** Comparison of genome statistics to other nematode species

Species	Phylogenetic clade	# Genes	CEGMA completeness	Average gene length	Assembly Length (bp)	# Scaffolds	N50		GC content %	Contig Length		
							#	Length		Mean	Median	Max Length
*Trichinella spiralis*	I	16,380	95.6%	952.8	63,525,422	6863	4	6,373,445	33.9	9256	1071	12,041,450
*Trichuris muris*	I	11,004	96.1%	1245.7	84,674,602	1683	59	400,602	44.8	50,312	1914	1,774,400
*Trichuris trichiura*	I	8813	96.1%	1293.9	75,496,503	4156	265	70,602	42.2	18,166	3965	533,758
*Ascaris suum*	III	15,260	98.9%	1188.5	265,545,801	31,538	260	290,558	37.8	8420	226	1,465,500
*Brugia malayi*	III	18,074	97.4%	1012.2	94,136,243	9827	62	191,089	29.6	9579	1340	5,235,760
*Dirofilaria immitis*	III	12,857	98.0%	1134.2	88,309,529	16,061	219	71,281	28	5498	754	1,085,577
*Loa loa*	III	15,445	97.8%	987.8	91,373,458	5773	130	174,388	31	15,828	1186	1,325,655
*Globodera pallida*	IV	16,403	83.8%	1079.6	124,672,549	6873	298	121,687	36.7	18,139	1699	600,076
*Meloidogyne hapla*	IV	14,420	97.2%	1044.7	53,017,507	3452	372	37,608	27.4	15,358	5814	360,446
*Caenorhabditis elegans*	V	30,697	100.0%	1229.5	100,286,401	7	3	17,493,829	35.4	14,326,629	15,279,421	20,924,180
*Haemonchus contortus*	V	24,466	95.0%	1124.8	369,846,877	23,860	1151	83,287	43.1	15,501	1515	947,606
*Necator americanus*	V	19,153	97.2%	804.6	244,075,060	11,864	284	211,861	40.2	20,573	1315	1,890,151
*Pristionchus pacificus*	V	24,217	98.0%	994.3	172,494,865	18,083	39	1,244,534	42.8	9539	685	5,268,024
AC-Orig	V	16,155	98.9%	894.3	348,994,891	8098	263	373,206	43.5	43,096	1515	2,174,208
AC-PB	V	17,540		962.8								

To evaluate and quantify improvement of genome annotation in our existing *Ancylostoma ceylanicum* assembly, we evaluated a long read mRNA library approach using PacBio single molecule real time (SMRT) technology. PacBio sequencing is unique when compared to sequencing-by-synthesis approaches. Read lengths are proportional to reaction times (movie lengths). Thus, PacBio is becoming the gold standard in long read sequencing technologies with average polymerase reads easily exceeding 14kbp (personal observations). As impressive as the read lengths are in SMRT sequencing, the single pass error rates are also impressively high (~ 15%; Table 2) dominated by insertions and deletions (indels). To compensate, a level of consensus accuracy (> 99%) is achieved with sufficient depth of coverage of long single molecules. In particular, a CCS is derived from multiple sequences from the same circular SMRTbell library template.

**Table 2:** P4 Chemistry Sequencing Statistics

Movie Length (mins)	Total Bases (MB)	Polymerase Reads		Reads of Insert		Zero Mode Waveguide Loading Efficiency		
		Length (bp)	Quality	Length	Quality	(P0)	(P1)	(P2)
75	409.5	5858	0.84	813	0.93	34,281 (23%)	69,907 (47%)	46,104 (31%)
75	432.28	5938	0.84	811	0.93	26,892 (18%)	72,796 (48%)	50,604 (34%)
75	408.76	5837	0.84	808	0.93	30,481 (20%)	70,029 (47%)	49,782 (33%)
75	398.05	6142	0.84	795	0.94	35,605 (24%)	64,808 (43%)	49,879 (33%)

In our work, we generated cDNA using total RNA isolated from the adult stage of the parasitic hookworm *Ancylostoma ceylanicum* (maintained in the Syrian Golden hamster *Mesocricetus auratus*). Hookworm infections are clinically considered a leading cause of iron deficiency anemia and protein malnutrition, primarily affecting children and pregnant women [4]. Two major hookworm species infecting humans, *Necator americanus* and *A. duodenale*, collectively infect an estimated 700 million people worldwide, predominantly in South and Central America, sub-Saharan Africa, and East Asia [5, 6]. However, recent epidemiological studies have identified *A. ceylanicum* as the next dominant hookworm species infecting humans following *N. americanus* in Asia [7–10]. The hamster-derived *A. ceylanicum* worms have long been utilized in studies of hookworm pathogenesis, vaccine development and anthelmintic testing since 1970s, due to the similar clinical features presented in hamsters when infected with *A. ceylanicum* as in human hookworm patients and the relative ease of obtaining samples [11–19].

To minimize the repertoire of over-abundant transcripts, we performed cDNA library normalization. In addition, we enriched transcripts based on the *A. ceylanicum* cDNA mode length (~ 1.3 kb) by size-selecting 2 kb PacBio SMRTbell libraries. In all, we generated over 270,000 reads represented by 192,288 CCSs as unique transcript molecules. We then supplemented the previously generated and publicly available Sanger based transcripts [20] with these newly obtained CCSs. To evaluate the improvement of the existing genome annotation, we re-ran the gene prediction pipelines against the existing genome assembly. Comprehensive comparison with the original annotated gene set provided deep understanding of the unique contribution of the PacBio mRNA sequences to the genome annotation.

## Results

### PacBio sequencing results

**Fig. 1:**
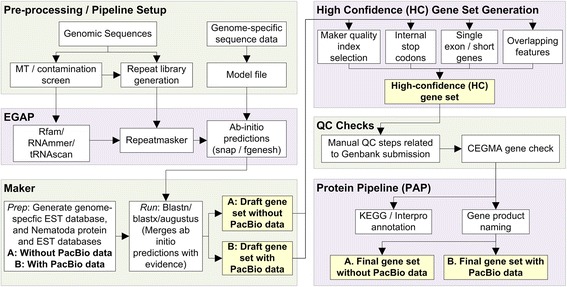
An overview of the gene prediction process. The gene prediction process, as described in the methods, is divided into major and minor steps

We used the *A. ceylanicum* genome assembly that we have generated (BioProject PRJNA72583) using a whole genome shotgun approach on the 454/Roche platform. Our ‘original’ *A. ceylanicum* genome annotation (“AC-Orig”, before the inclusion of PacBio data; BioProjectID # PRJNA72583, GenBank uploaded in May 2013), contained 16,026 predicted genes, and used 10,591 available *A. ceylanicum* EST sequences (spanning the L3i larva and adult life cycle stages) downloaded from the NCBI database. The AC-Orig annotation and the annotation using the PacBio data (AC-PB) was done on the same genome assembly using the same annotation pipeline (*see* Methods; Fig. 1).

To improve upon the AC-Orig annotation, we primed total RNA using the SMARTer technology with the goal of polymerizing long 1st-strand cDNA molecules that possess a common 5′ and 3’ PCR priming site for subsequent single primer PCR amplification. This single primer technique only amplifies 1st-strand cDNAs synthesized with both priming sites during the RT reaction, and the amplification products are typically > 500 bp minimizing the representation of smaller cDNA molecules [21]. This is the strategy PacBio uses for their Isoform Sequencing (Iso-Seq) protocol.

Unlike the Iso-Seq protocol, we added a cDNA normalization step to our protocol since each SMRT Cell consists of only 150,000 zero-mode waveguides (ZMWs) with single molecule loading efficiencies in the 30–50% range. Normalization is one method to minimize overabundant cDNA molecules synthesized from highly expressed transcripts, allow detection of under-represented transcripts, and reduce costs by minimizing the number of SMRT Cells required for data generation. The normalized cDNA length distribution was assessed on the Agilent BioAnalyzer, 2100 (Agilent Technologies, Cedar Creek, Texas; default settings) and the electropherogram revealed cDNA molecules ranged from 500 bp to slightly greater than 2 kb. Using this cDNA source, the generated 2 kb PacBio SMRT-bell library was subsequently sequenced on the Pacific Bioscience RS.

The normalized, male adult stage *A. ceylanicum* cDNA SMRTbell library was sequenced using the P4 polymerase across four SMRT Cells and imaged for 75 min (Table 2). This generated 124 million bases from 192,888 CCS with a maximum read length of 3572 bp and a mean CCS read length of 730 bases. Overall, the normalized library produced an additional 124 Mbp of unique data from the same RNA source that was also used to generate Illumina RNA-Seq data. The Illumina RNA-seq data was used both at: a) raw RNA-seq reads level, and b) assembled Illumina transcripts level, to compare and orthogonally validate the AC-Orig and AC-PB annotations.

### General features of the revised gene prediction

The newly obtained CCSs, combined with previously downloaded EST sequences [20] were used as transcriptomic evidence and provided to our *in-house* gene prediction pipeline, consisting of RNA predictors, a combination of ab initio and evidence-based predictors and Maker (Fig. 1).

Our new gene set utilizing the PacBio data (AC-PB) contained 17,540 protein-coding genes, including 1609 genes (9.2%) that did not overlap with the previously identified with the original annotation (AC-Orig) (Table 3). Approximately 47% (8238) of the AC-PB genes were mapped by nearly 75% (143,666) of the PacBio CCSs, with 50.4% (811) of the AC-PB unique genes being “expressed” (covered by at least 50% of their length). While the number of AC-Orig and AC-PB genes considered to be expressed based on the coverage with the Illumina RNA-seq reads (42 million RNA-seq reads) was similar (64.9% vs. 66.8%, respectively), more of the AC-PB-unique genes were expressed (1356, 84.3%) than AC-Orig-unique genes (151, 69.3%).

**Table 3:** Illumina and Pacbio RNA-Seq read coverage over predicted gene sets

Read Type	Gene Set	Subset of genes	Total count	# of expressed genes (breadth ≥50%)	% expressed	Average read depth	
						Any	Expressed
Illumina Reads	AC-Orig	All genes	16,026	10,405	64.9%	161.1	245.6
		Overlapping AC-PB genes	15,808	10,254	64.9%	160.9	245.5
		Not overlapping AC-PB genes	218	151	69.3%	171.3	246.4
	AC-PB	All genes	17,540	11,721	66.8%	156.3	231.6
		Overlapping AC-Orig genes	15,931	10,365	65.1%	158.3	240.8
		Not overlapping AC-Orig genes	1609	1356	84.3%	136.5	161.1
	Schwarz et al., [31]		36,687	16,376	44.6%	90.4	199.4
PacBio Reads	AC-Orig	All genes	16,026	3166	19.8%	3.3	8.9
		Overlapping AC-PB genes	15,808	3128	19.8%	3.2	8.8
		Not overlapping AC-PB genes	218	38	17.4%	4.1	12.8
	AC-PB	All genes	17,540	4209	24.0%	3.6	8.8
		Overlapping AC-Orig genes	15,931	3398	21.3%	3.4	8.7
		Not overlapping AC-Orig genes	1609	811	50.4%	6.4	9.1
	Schwarz et al., [31]		36,687	4903	13.4%	2.1	8.8

**Fig. 2:**
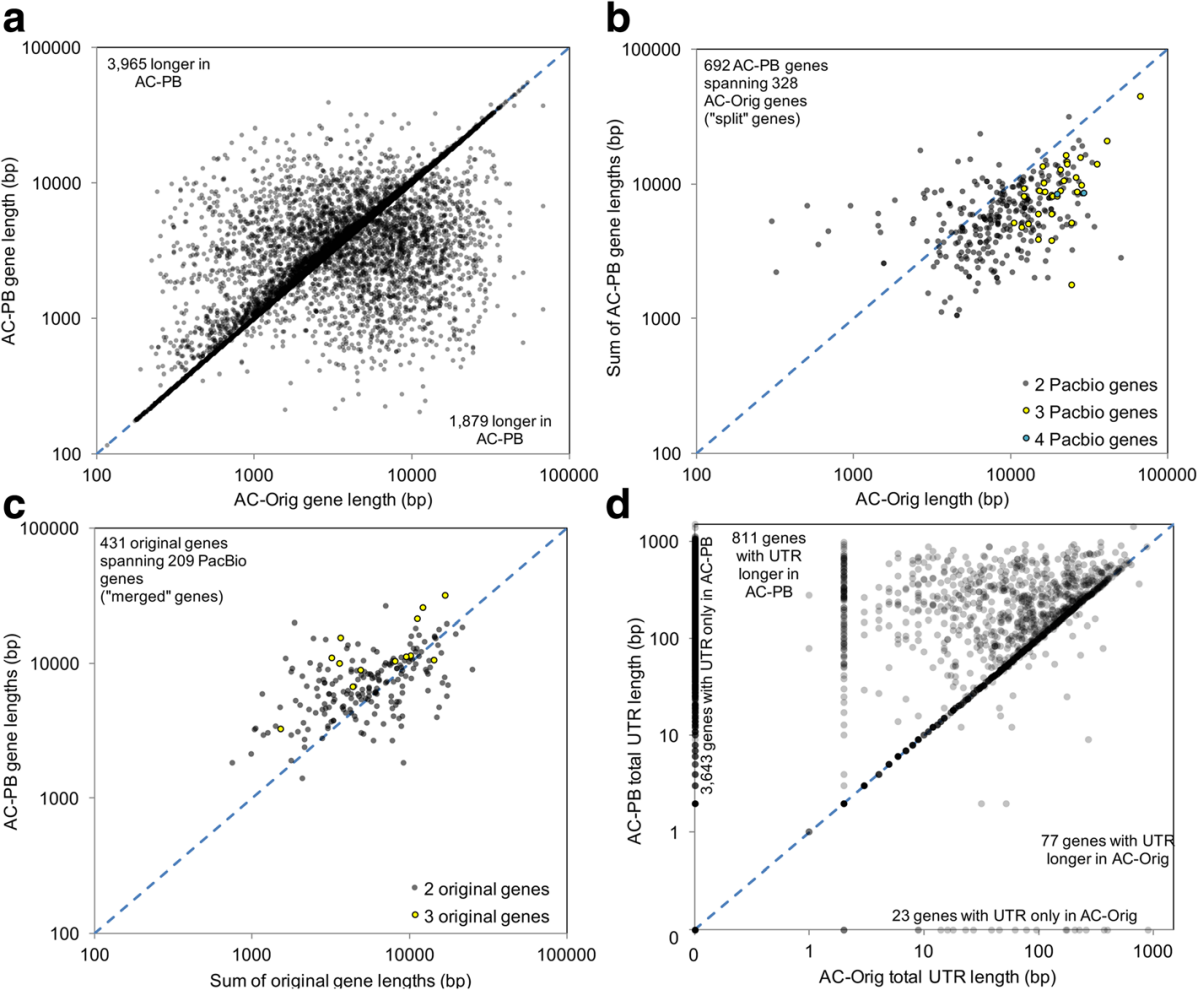
Differences in gene lengths and UTR lengths between AC-Orig and AC-PB. Differences in gene lengths are shown for: **a** Genes not split or merged between the annotations, (**b**) Genes split in AC-PB compared to AC-Orig, and (**c**) Genes merged in AC-PB compared to AC-Orig. **d** Differences in UTR lengths (summed for 5′ and 3′) between AC-Orig and AC-PB. Additional file 1 shows the UTR lengths separate for 5′ and 3′ regions

**Fig. 3:**
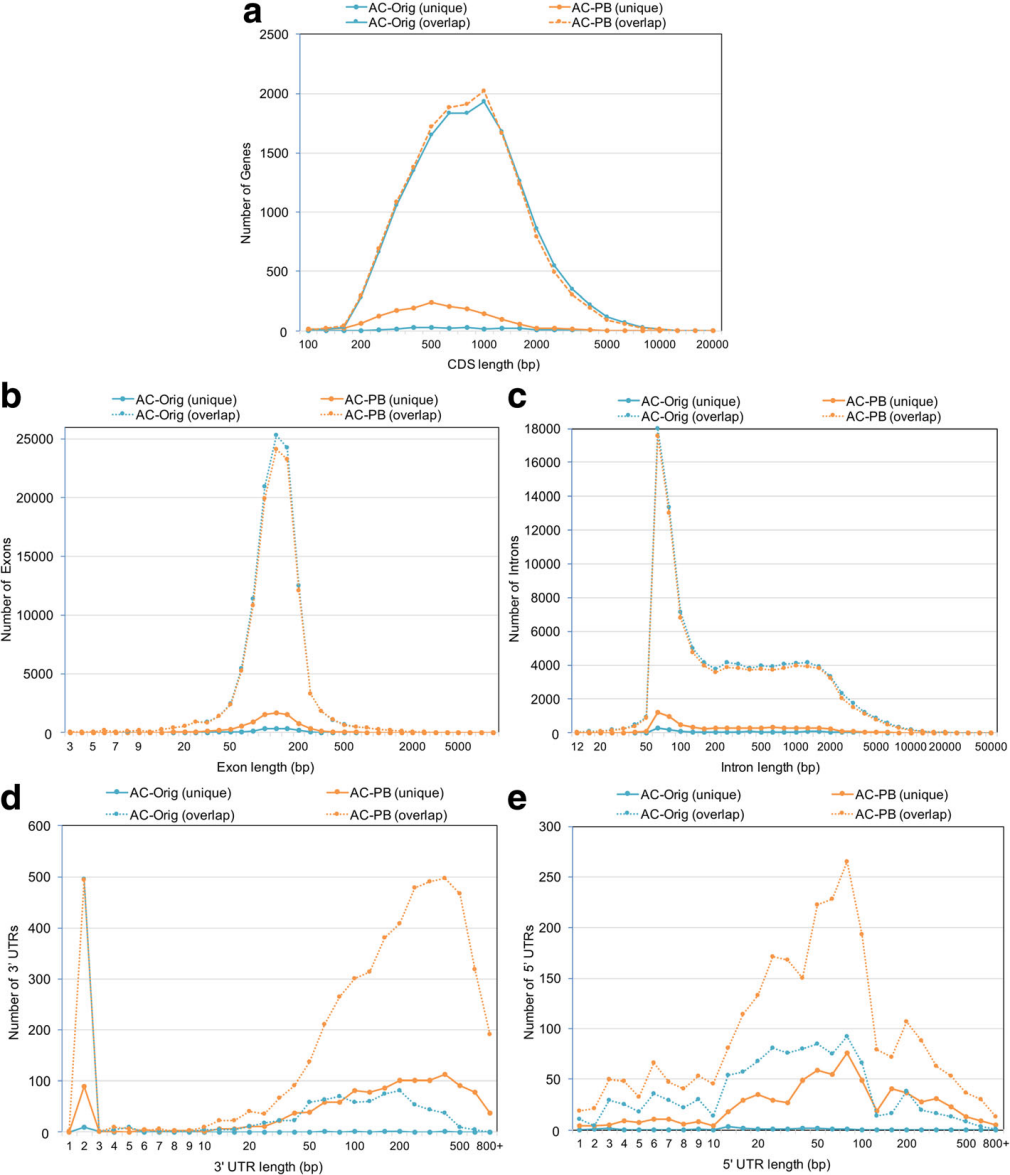
Frequency distribution plots for AC-Orig (blue) and AC-PB (orange) for (**a**) CDS Lengths, (**b**) Exon lengths, (**c**) Intron lengths, (**d**) 3’ UTRs and (**e**) 5’ UTRs

**Fig. 4:**
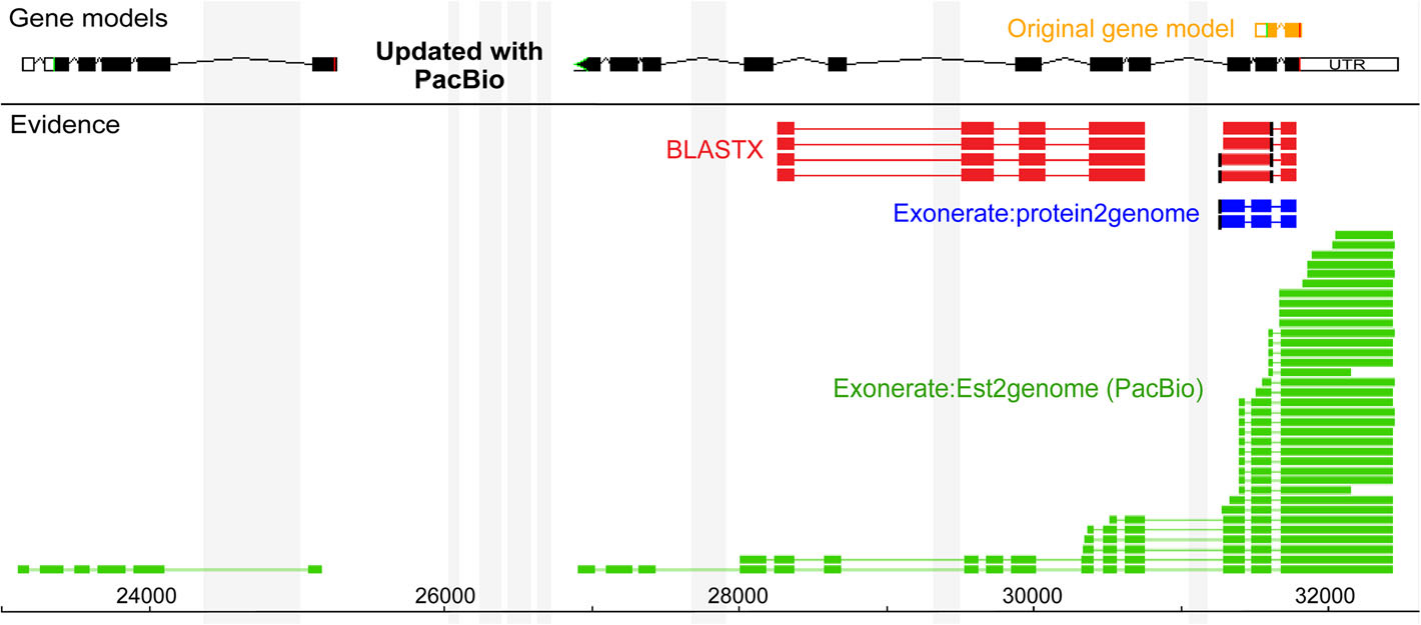
An example of an improved gene structure predictions in AC-PB (black) compared to AC-Orig (orange). The BLASTX (red) and protein2genome (blue) predictions used for AC-Orig predicted a short gene model, but additional PacBio evidence (green) extended the existing gene, and also predicted a second gene at the 5′ end of the original gene. Shaded grey areas represent masked repeat sequences in the assembly

Of 15,931 AC-PB genes for which the loci overlapped > 10% with those in AC-Orig, 3965 had increased length (Fig. 2a), while only 1879 decreased in length. The AC-PB genes overlapping the AC-Orig genes were significantly shorter on average (4686.6 bp vs. 5068.1, *P* = 0.02 by two-tailed T-test with unequal variance; Shapiro Wilk W = 0.96, using Log values). Additionally, 328 AC-Orig genes were split into two to four AC-PB genes (692 total; Fig. 2b), and 431 AC-Orig genes were merged into 209 AC-PB genes (Fig. 2c). The new AC-PB predictions represent a 1.9 Mb increase in CDS / exon length compared to AC-Orig (totaling to 7.92 Mb of genomic sequence; Figs. 3a and 1b), although this was accompanied by a 2.2 Mb decrease in intron length (Fig. 3c). Figure 4 demonstrates a specific example in which PacBio reads expand the length of a gene and predict a new gene, where no evidence existed before, within the same genomic region. In addition, the AC-PB-unique genes contained a total of 162,659 bp and 204,894 bp of CDS not covered by Illumina reads or assembled Illumina transcripts (respectively) compared to 67,787 and 77,403 for the AC-Orig unique genes. This resulted in 6.3% and 16.0% of AC-PB genes not being covered at all by Illumina reads or assembled Illumina transcripts at all (respectively; Table 4). In addition, 20.1% (2934) of all assembled Illumina transcripts had no overlap with genes from either assembly (“Illumina-unique”). Of those, 7.8% (228/2934) had no hits in the NCBI NR database, compared to only 0.05% (56/11,677) for the assembled Illumina transcripts with hits to either gene set. Among the transcripts with hits in NCBI, the average log E value of the Illumina-unique transcripts was significantly higher (i.e. a weaker match) than those that overlapped genes (*P* < 10^− 10^, two-tailed Mann-Whitney U test), and their average length was also significantly shorter than those that overlapped genes (765.0 vs 1539.5; *P* < 10^− 10^, two-tailed Mann Whitney U test).

**Table 4:** Summary of CDS and 5′ and 3’ Untranslated Region (UTR) statistics, including overlaps between gene sets and with assembled Illumina transcripts and RNAseq Illumina reads

Gene region	Statistic		AC-Orig			AC-PB		
			All genes	Overlapping genes	Unique genes	All genes	Overlapping genes	Unique genes
CDS	# of genes		16,026	15,808	218	17,540	15,931	1609
	Average length (bp)		962.8	962.4	994.3	894.3	922.0	619.8
	Total length (kbp)		15,430.0	15,213.2	216.8	15,685.3	14,688.0	997.3
	Coverage by Illumina reads	% of genes	78.1%	78.0%	80.7%	79.6%	78.2%	93.7%
		% of bases	66.9%	66.8%	68.7%	67.5%	66.4%	83.7%
		Length not covered (kbp)	5111.7	5043.9	67.8	5104.8	4942.2	162.7
	Coverage by Illumina Stringtie contigs	% of genes	65.2%	65.1%	67.9%	66.9%	64.8%	84.0%
		% of bases	61.8%	61.8%	64.3%	62.4%	61.2%	79.5%
		Length not covered (kbp)	5895.8	5818.4	77.4	5899.6	5694.7	204.9
5’ UTR	# of genes		1101	1083	18	3404	2702	702
	Average length (bp)		57.7	58.2	24.3	88.2	83.9	104.8
	Total length (kbp)		63.5	63.1	0.4	300.2	226.7	73.6
	Coverage by Illumina reads	% of genes	74.1%	74.1%	72.2%	78.7%	79.8%	74.2%
		% of bases	61.5%	61.3%	89.7%	73.1%	74.7%	68.4%
		Length not covered (kbp)	18.7	18.6	0.1	80.7	57.4	23.3
	Coverage by Illumina Stringtie contigs	% of genes	77.3%	77.2%	83.3%	79.5%	80.4%	76.2%
		% of bases	70.6%	70.5%	86.7%	66.8%	69.2%	59.5%
		Length not covered (kbp)	24.5	24.4	0.0	99.5	69.8	29.8
3’ UTR	# of genes		1234	1218	16	6608	5363	1245
	Average length (bp)		78.4	78.8	52.3	232.1	234.7	221.0
	Total length (kbp)		96.8	95.9	0.8	1533.9	1258.8	275.1
	Coverage by Illumina reads	% of genes	50.1%	50.3%	31.3%	73.3%	74.6%	67.5%
		% of bases	73.9%	73.9%	75.6%	77.0%	78.2%	71.2%
		Length not covered (kbp)	31.1	30.8	0.3	513.6	402.5	111.1
	Coverage by Illumina Stringtie contigs	% of genes	56.3%	56.7%	25.0%	70.4%	71.5%	65.3%
		% of bases	67.9%	67.9%	60.0%	66.5%	68.0%	59.6%
		Length not covered (kbp)	25.3	25.1	0.2	353.1	273.9	79.2

In addition to increased coding content obtained in the presence of the PacBio long cDNA CCSs, the AC-PB data set demonstrated a fifteen-fold increase in non-coding representation, primarily from an increase in UTRs (Fig. 2d; Table 5; Additional file 1). The largest increase was observed in the base representation of the 3’ UTRs (Additional file 1, panel a), with an overall increase of 1.4 Mb and a total representation of 1.8 Mb, but there was also a large improvement in 5’ UTR representation (Additional file 1, panel b), and improved non-coding representation, from 40 kb to greater than 270 kb. Additionally, the number of genes with 3’ UTRs and 5’ UTR increased by 5-fold and 3-fold (respectively), with a nearly 2-fold increase in overall UTR lengths (Fig. 2d, Table 5), and a significant increase in average identified 3’ UTR length (233.2 bp in AC-PB vs 81.0 bp in AC-Orig, *P* < 10^− 10^ by Mann-Whitney U test; Fig. 3d) and 5’ UTR length (88.2 bp in AC-PB vs 57.7 bp in AC-Orig, P < 10^− 10^ by Mann-Whitney U test; Fig. 3e). In the genes exclusive to only one annotation or the other, there were far more AC-PB unique genes with 5’ UTRs (702) than AC-Orig unique (18), and these were also significantly longer (average of 104.8 bp vs 24.3 bp for AC-Orig unique, *P* = 5 × 10^− 5^). Overall, 23,279 bp and 29,761 bp of the AC-PB unique genes were not covered by Illumina RNA-seq reads or assembled Illumina transcripts (respectively, compared to just 58 bp and 48 bp for AC-Orig unique genes). This resulted in 25.8% and 23.8% of AC-PB unique genes with 5’UTR having no Illumina coverage whatsoever (Table 5).

**Table 5:** A Summary of characteristics of assemblies annotated without and with PacBio mRNA sequences

Statistic	Original *A. ceylanicum* genome annotation (AC-Orig)	Improved *A. ceylanicum* genome annotation using PacBio data (AC-PB)	AC-Orig genes overlapping (10%) AC-PB genes with PacBio evidence	AC-PB genes with PacBio evidence (with or without EST evidence)
Number of genes	16,026	17,540	6734	8238
Number of single exon genes	805	863	154	211
Total length of all exons (bp)	15,590,301	17,519,546	7,915,169	9,931,507
Total number of exons	117,877	121,578	63,273	67,714
Average exon length (bp)	132.3	144.1	125.1	146.7
Average # exons/gene	7.4	6.9	9.4	8.2
Total length of all CDS exons (bp)	15,429,981	15,685,322	28,877,304	27,490,435
Total number of CDS exons	117,657	119,866	63,129	66,083
Average CDS exon length (bp)	131.1	130.9	123.5	123.2
Average # coding exons/gene	7.3	6.8	9.4	8.0
Total length of all introns (bp)	63,133,642	60,868,345	28,930,431	28,142,643
Total number of introns	101,851	104,038	56,566	59,476
Average intron length (bp)	621.2	594.9	511.7	473.2
Average # introns/gene	6.4	5.9	8.4	7.2
Total UTR length (bp)	160,320	1,834,224	117,930	1,788,957
Number of genes with UTR	1889	7295	1205	6567
Average size of UTR per gene with UTR	84.9	251.4	97.9	272.4
Number of genes with UTR < 10 bp	1228	3966	744	3451
Number of genes with UTR 10 bp - 100 bp	423	1058	287	908
Number of genes with UTR > 100 bp	238	2271	174	2208
Total 5’ UTR length (bp)	63,556	300,333	39,954	273,401
Number of genes with 5’ UTR	1150	3488	710	3013
Number of genes with spliced 5’ UTR	127	745	82	696
Total 3’ UTR length (bp)	96,764	1533,891	77,976	1515,556
Number of genes with 3’ UTR	1238	6611	817	6145
Number of genes with spliced 3’ UTR	45	400	33	388
# of ESTs at 3’	–	–	3610	105,053
polyA signal ‘aataaa/attaaa’	–	–	1307	61,069
polyA signal ‘agtaaa’ only	–	–	215	7775
polyA total (any signal)	–	–	1522	68,844

We observed that 3’-UTRs resided mostly (79% for AC-Orig and 97% for AC-PB) within a single exon flanking the CDS, but the number of 3’ UTRs containing spliced exons were significantly higher in the AC-PB gene set (400 vs 45 in AC-Orig). For non-spliced UTRs, the lengths of exons where UTRs resided were longer in AC-PB (1,504,453 compared to 124,243 for AC-Orig) and in higher number (6165 compared to 1684 for AC-Orig). For overlapping genes with UTR exons, the UTRs were longer on average in AC-PB for both the 3’ UTR exons (224.0 bp vs 76.4 bp in AC-Orig, P < 10^− 10^ by Mann-Whitney U test) and the 5’ UTR exons (45.8 bp vs 35.9 bp in AC-Orig, *P* = 1 × 10^− 6^). In the genes exclusive to only one annotation or the other, there were far more AC-PB unique genes with 3’ UTRs (1245) than AC-Orig unique (16), and these were also significantly longer (average of 221.0 bp vs 52.3 bp for AC-Orig unique, P = 5 × 10^− 6^). Overall, 111,123 bp and 79,177 bp of 3’ UTRs from AC-PB unique genes were not covered by any Illumina RNA-seq reads or assembled Illumina transcripts (respectively, compared to just 334 bp and 204 bp for AC-Orig unique genes). This resulted in 32.5% and 34.7% of AC-PB unique genes with 3’UTR having no Illumina coverage whatsoever (Table 5). Overall, AC-PB genes identified 0.51Mbp and 0.35Mbp of 3’UTR not covered by Illumina reads or assembled transcripts (respectively). These findings show that the UTR length changes observed in AC-PB can be attributed to both an increased number of identified UTR exons as well as increased lengths of the existing UTR exons.

### Increased representation of full-length transcript by CCSs

The increased number of 5′/3’ UTRs identified in AC-PB suggested that transcriptomic evidence supplemented with PacBio CCSs provides additional information useful for annotating full-length transcripts. Sequence analysis revealed that 15,993 PacBio CCSs contained complete open reading frames (ORFs) and corresponding 5′/3’ UTRs for 2800 genes, while AC-Orig contained complete ORFs and UTRs for only 239 genes.

We also extracted UTR features from full-length *A. ceylanicum* transcripts and searched them for 22-nt spliced leader sequences (SLs). SL1 is conserved across nematode species, and genes with SL1 have previously been cloned from *A. ceylanicum* [22, 23]. In addition to SL1s, polyA signals (PASs) are a feature of eukaryotic protein-coding genes, defining the transcription termination site. Most PASs are conserved with sequence motifs of “AATAAA” or the close variant “ATTAAA” [24, 25]. We obtained all the ESTs/PacBio CCSs that were aligned to the 5′/3’-UTRs, and then performed the direct searches of the 5’ SL sequences and 3’ PASs on those ESTs/PacBio CCSs. We found that 18,115 and 101,684 of our long RNA-Seq CCSs were aligned to the 5’-UTRs of 3765 genes and 3’-UTRs of 7321 genes (respectively). In contrast, only 1729 and 3369 downloaded ESTs were aligned to the 5′ and 3’ UTRs of predicted *A. ceylanicum* genes, (respectively), further suggesting the importance of PacBio CCSs in UTR identification. Among all the PacBio CCSs aligned to 5’ UTRs, 115 (corresponding to 108 genes) contained SL1 sequences, and 19 (corresponding to 18 genes) contained SL2 sequences. Using the AC-PB data, we were able to identify a base switch at positions 15/16 in SL2 compared to *C. elegans*, which is a novel finding since the SL2 sequence has never previously been reported for *A. ceylanicum* (*C. elegans*: GGTTTTAACCCAGTTACTCAAG, *A. ceylanicum*: GGTTTTAACCCAGTATCTCAAG). Similarly, PASs in 3’-UTRs were much more highly represented in PacBio CCSs (49,926 CCSs / 4086 genes) compared to ESTs (538 ESTs). PASs detected with PacBio had a mean distance to the stop codon of 214 nt. One striking feature was the low number of polyA/polyT sequences present in 3′ end CCSs (1662 out of 49,926 CCSs).

### Gene sets annotated with PacBio reads have increased overall functional potential

Functional analysis of the annotated gene sets was performed by searching the KEGG database (Additional file 2) [26]. The results showed an increase KEGG matches in the AC-PB predictions (15,226 genes, 86.8% of AC-PB genes) when compared with AC-Orig (12,995 genes, 81.1% of AC-Orig genes), as well as an increase in the number of unique KEGG orthologous groups identified (3923 vs 3711, respectively; 3600 in common). The most expanded KEGG pathway was the broad parent category “Metabolic Pathways” (ko1100), with 36 (5.8%) more KEGG Orthologous group classifications (KOs) and 291 more genes (13.7%) annotated to this pathway in AC-PB compared to AC-Orig. The second most expanded pathway among AC-PB was “Biosynthesis of secondary metabolites” (13 additional KOs and 89 additional genes), which is an important pathway for studying signaling between parasitic nematodes and hosts, as well as with their symbiotic bacteria [27–29]. Another strongly expanded pathway, “MAPK signaling” (ko4010) (2 additional KOs and 41 additional genes; 19.4% increase) is important for environmental signaling and stress responses in nematodes [30]. Counts of KOs and genes per pathway are shown in Additional file 2.

## Discussion

To facilitate a better understanding of hookworm biology, tracking hookworm infection history and distribution, and eventually developing effective intervention strategies that can be applied to all the hookworm diseases, determining their genome information is a prerequisite. In addition to the de novo genome assembly of *A. ceylanicum* presented here, we have also published a de novo genome assembly of *N. americanus* with 19,542 annotated genes [1]. Annotating these hookworm genomes has been challenging due to insufficient transcriptomic evidence obtained either using the second-generation 454/Roche pyro-sequencing or Sanger sequencing, and only recently Illumina platform. The Sanger sequencing is labor-intensive and low-throughput, even though the generated reads are long (~ 750 bp) and with high accuracy. 454/Roche-pyro sequencing is relatively cost-effective and high-throughput, but the reads are typically short, with high base-composition bias. Therefore, the resultant hookworm genome drafts and annotated genes were often missing start/stop codons and un-translated region (UTR) annotations. In addition, evidence of long non-coding RNAs (lncRNAs) and transcript isoforms are either undefined or underestimated. Complex eukaryotic genome assemblies and annotations required time and effort to be generated and many of them never get improved overtime. Providing a detailed quantification of the level of improved annotation using different mRNA sequencing technologies without re-sequencing the genome provides valuable information to make an informed decision.

With the exceptional SMRT sequencing technology introduced by PacBio, we generated the deepest and the longest long-read dataset to date for the *A. ceylanicum* transcriptome. These long PacBio CCSs were used to complement the existing ESTs (AC-Orig) and a revised *A. ceylanicum* gene prediction (AC-PB) was obtained. The results showed that 92.2% of the PacBio CCSs (177,843/192,888) were mapped to the *A. ceylanicum* genome (94.2% mapped to the published version of the genome [31]), indicating the consensus sequences are of high quality. The revised gene predictions revealed 2 MB of new coding regions and identified 1609 new genes when compared with the previous version AC-Orig which only used available ESTs as transcriptomic evidence.

In addition to the merging of many split genes, one of the distinctive features of AC-PB in comparison with AC-Orig was the increase in number and length of UTRs, particularly 3’ UTRs, clearly demonstrating the advantage of long CCSs in defining gene UTRs and consequently more complete ORFs. Compelling evidence has shown that UTR regions correlate with the complexity of gene expression regulation in eukaryotic organisms [32], furthering the importance of PacBio platform in gene discovery and identifying gene boundaries with increased number of UTRs and UTRs containing splicing sites. Here, we also identify 134,390 bp of UTR among genes only identified by PacBio annotation which were also not identified by Illumina RNAseq reads, further highlighting the importance of PacBio sequencing annotation in comprehensive UTR annotation. These results were consistent with the common perception that an individual PacBio CCS from our SMARTer-SMRT library would represent all the information originating from a single RNA molecule.

Although PacBio CCSs significantly improved gene predictions, sequences at the end of the transcripts were still missing, as evidenced by low detection rates of SLs and polyA tails. Possible explanations for this phenomenon include incomplete transcripts due to minor RNA degradation based on the 8.9 RIN value, or that we did not specifically enrich for 5’ mRNAs using a cap-trapping strategy. Here, we relied on the ability of the SMARTer strand-displacement method to provide 5′ transcript information and carefully highlight this is a long (not a full-length) cDNA method as opposed to the Iso-Seq full-length claims. This approach highlights near full-length transcript representation [33]. We also noticed that some polyAs were present in the middle of PacBio CCSs, suggesting possible lysine-rich structural genes as associated with basic proteins or potentially inappropriate joining of two transcript fragments. Meanwhile, a strong bias toward the 3’ UTR identification existed, which is a result from the oligo dT priming resulting in cDNAs representing truncated transcripts due to RNA degradation or inefficiency during the reverse transcription reaction. To improve on the 5’ UTR representation in our long cDNA protocol, one could add an additional cap-trapping strategy enriching for 5’ mRNA transcript representation. Other expected improvements could come due to use of a new chemistry (beyond C4) or moving to other PacBio platforms (RSII vs. Sequel).

## Conclusions

Overall, our normalized, long cDNA method generated > 190,000 CCS, and our pipeline improvements identified 1609 (9.2%) new *A. ceylanicum* genes, extended the length of 3965 (26.7%) genes and increased the total genomic exon length by 1.9 Mb (12.4%). Non-coding sequence representation (primarily from UTRs) was particularly improved, increasing in total length by fifteen-fold, by increasing both the length and number of UTR exons. In addition, the UTR data provided by PacBio reads allowed for the identification of a novel SL2 splice leader sequence for *A. ceylanicum* and an increase in the number and proportion of functionally annotated genes. In conclusion, PacBio data has supported a significant improvement in gene annotation in this draft genome, and is an appealing alternative or complementary approach to annotation obtained by using other transcript sequencing technologies.

## Methods

### *A. ceylanicum* collection and preparation

An Indian strain of *A. ceylanicum* (US National Parasite Collection No. 102954) was maintained in Syrian hamsters (*Mesocricetus auratus*) as described previously [13]. All animal experiments were carried out in strict accordance with the recommendations in the Guide for the Care and Use of Laboratory Animals of the National Institutes of Health and under protocols approved by the George Washington University Institutional Animal Care and Use Committee. Hamsters with mature infections (greater than 21 days) were euthanized by CO2 overdose, and the small intestine removed, split longitudinally, and incubated in RPMI medium for 1–2 h to allow the adult worms to detach from the intestinal wall. The worms were collected by hand, washed 5 times with sterile RPMI medium, snap-frozen by immersion in liquid nitrogen, and stored at -80C until used for nucleic acid isolation.

Infective third-stage larvae (iL3) were recovered from coprocultures after approximately one week at 27 °C by modified Baermann technique and stored up to 4 weeks in BU buffer (50 mM Na2HPO4/22 mM KH2PO4/70 mM NaCl, pH 6.8) [34] at room temperature prior to infection. A male hamster was infected with approximately 80 *A. ceylanicum* iL3 by oral gavage. At day 16 post infection (PI), the hamster was sacrificed and the small intestine opened longitudinally in PBS kept at 37 C on a slide warmer for several hours until the worms released from the intestinal wall. The worms were individually transferred to a dish containing warm PBS using a transfer pipette and incubated for 30–60 min to allow them to disgorge ingested host tissue. The worms were individually transferred to a second dish for 30 min, then picked into a microcentrifuge tube containing PBS. The worms were washed by vortex and allowed to settle, and the liquid removed. The washes were repeated until any remaining host tissue was removed. The worms were snap frozen in liquid nitrogen and stored at -80 C until used for RNA extraction.

Total RNA was extracted from ten 16 day PI male *A. ceylanicum* using Trizol (Thermo Fisher) according to the manufacturer’s instructions. Following precipitation, the RNA was resuspended in nuclease free water and treated with DNAse I (Thermo Fisher) for 15 min at 22C. To stop the reaction, 2.5 mM EDTA was added and the reaction heated at 65 C for 10 min.

### *A. ceylanicum* cDNA preparation

As input into our long cDNA protocol, we started with 450 ng of DNase-treated total RNA prepared from *A. ceylanicum* with a corresponding RNA integrity number (RIN) of 8.9. We followed the SMARTer PCR cDNA Synthesis Kit protocol (Clontech Laboratories, Inc., Mountain View, CA; #634925), briefly highlighted: first-strand cDNAs synthesis was performed by oligo dT annealing and reverse transcription at 42 °C for 90 min. During this incubation, terminal-transferase activity associated with the reverse transcriptase sequentially adds 3′-cytosines onto the polymerized first strand. This poly-cytosine overhang anneals with 3′-guanosines as part of the 5’ SMART IIA oligo (included in the RT reaction). Template switching during the RT reaction extends the first strand cDNA and incorporates the complement of the 5’ SMART IIA oligo. Depending on the condition of the RNA, this system may generate full-length cDNAs as well as nearly full-length (long) cDNA molecules. These long first-stranded cDNA molecules are subsequently amplified during single primer PCR amplification enriching for product sizes > 500 bp [21] Primers are as described in the Smart IIA documentation (Clontech Laboratories, Inc., Mountain View, CA; #634925).

Prior to single primer amplification, PCR optimization was used to minimize potential amplification artifacts with the long cDNAs. We added 1 μL of 1st Strand cDNA with SYBRFAST Universal 2X qPCR Master Mix (Biosystems, Inc., Woburn, MA.) and 200 nM primer IIA 5’-AAGCAGTGGTAACAACGCAGAGT. The cDNA was amplified with the Eppendorf epigradient S qPCR instrument (98° 2 min, 30 cycles of 98° 10 s 65 °C 30 s). The optimal PCR cycle threshold (Ct) for long cDNA amplification was determined using the RealPlex 2.2 software (Eppendorf). The Ct value was 14 cycles. We performed sixteen independent cDNA amplification reactions using 25 μl KAPA 2× HiFi HotStart Ready mix, 1 μl 1st Strand cDNA, and 200 nM Primer IIA. We used the following cycling conditions: 5 min at 95 °C, followed by 14 cycles of 30 s at 95 °C, 30 s at 65 °C, and 3 min at 68 °C. The PCR-amplified second-strand cDNA was concentrated using QIAquick PCR Purification Kit column (Qiagen Inc., Valencia, CA).

To minimize the potential representation of over abundant transcripts and reduce the required number of SMRT Cells for unique transcript identification, we normalized the cDNA using the Trimmer-2 cDNA normalization kit following the manufacture’s instruction (Evrogen JSC, Moscow, Russia). In brief, 300 ng aliquots of cDNA was denatured for 2 min at 98 °C followed by hybridization for 5 h at 68 °C under mineral oil. The re-annealed second-strand cDNA in each aliquot was digested for 10 min at 68 °C with 1 U of Trimmer duplex-specific nuclease (DSN) to obtain the normalized cDNA. The mineral oil was removed and the samples were combined. The normalized cDNA was again subject to PCR amplification as described above for 14 cycles. The amplified product was used as input into PacBio SMRTbell library construction.

### PacBio library construction and sequencing

Duplicate 2 kb PacBio SMRTbell libraries were prepared, each from 750 ng un-fragmented, normalized cDNA, according to the DNA Template Prep kit 2.0 protocol (250 bp-3 kb) (Pacific Biosciences, Menlo Park, CA, USA, #100–222-300). The PacBio library complex was mixed with DNA/Polymerase binding kit P4 (4.5:1,v:v) (Pacific Biosciences, Menlo Park, CA, USA, #100–236-500) to obtain an on-plate concentration of 0.225 nM. Samples were sequenced at Washington University’s HHMI-designated Pacific Biosciences (PacBio) sequencing site, which is equipped with RSII technology, capable of generating an average of 4–6 Kb read length with 3–20 Kb library preparations, generating about 1200 Mb per SMRT cell. The library complex was sequenced with four SMRT cells at 75-min collection protocol (Standard Seq v3) and C2 sequencing chemistry, producing 192,888 CCSs.

### Genome sequencing and assembly

The *A. ceylanicum* whole genome shotgun libraries with 3 kb and 8 kb inserts were prepared and sequenced on the Roche/454 platform, followed by Newbler assembly, as previously described [2, 35]. The initial assembly was further improved using our in-house tools CIGA (Cdna tool for Improving Genome Assembly) and PyGap (gap closure tool) by incorporating 1.56 million 454 cDNA reads from the closely-related hookworm *A. caninum* [36] that linked different assembly contigs. The Pyramid assembler (packaged in PyGap) was then used to align Illumina genomic paired-end reads to extend contigs and close gaps.

### Gene calling, annotation and comparison

Repeat sequences were identified by generating a custom repeat library using Repeatmodeler (http://www.repeatmasker.org/RepeatModeler/). The ribosomal RNA genes were identified using RNAmmer [37] and transfer RNAs (tRNAs) were identified using tRNAscan-SE [38]. Other non-coding RNAs (such as microRNAs) were identified by a sequence homology search of the Rfam database [39]. These repeats and predicted RNAs were then masked using RepeatMasker [40]. Protein-coding genes were predicted using a combination of ab initio predictors Snap [41] and Fgenesh [42] and the evidence based predictor Augustus [43]. These predictions were fed to the annotation pipeline tool Maker (version 2.26) [44] which utilizes aligned EST [20] and protein evidence, to revise the predicted gene structures. A consensus gene set from the above prediction algorithms was generated, using a logical, hierarchical approach developed at The McDonnell Genome Institute [1]. In summary, Quality Index (QI) values produced by Maker were evaluated to produce a high confidence gene set, by retaining gene predictions containing (a) splice sites confirmed by an EST or exons that overlap an EST, or (b) exons that overlap multiple ESTs or protein alignments were retained. The remaining genes were retained as part of the final gene set if they met at least one of the following criteria: (i) A significant BLAST against Swissprot [45] (E < 1e-6); (ii) A significant RPSBLAST against Pfam [46] (E < 1e-3); (iii) A significant RPSBLAST against CDD [47] (E < 1e-3 and coverage > 40%) or (iv) A significant similarity-based search against GenesDB from KEGG [26, 48], (≥55% identity and ≥35 bit score). No genes with > 10% overlap were retained in the final gene set, and limited manual review was performed to confirm the core gene set used to evaluate completeness (CEGMA [49, 50])**.** This process was repeated utilizing the PacBio reads as evidence during the Maker gene annotation.

Functional annotations of the deduced proteins were determined using a BLAST search against the KEGG database [26, 48] to assign enzyme-based biological pathways annotations. Gene product naming was determined by BER (JCVI: http://ber.sourceforge.net).

### Illumina RNA-seq library construction, sequencing, and processing

Non-normalized cDNA from a 16-day adult male whole-worm *A. ceylanicum* sample was used to construct Multiplexed Illumina paired end small fragment libraries according to the manufacturer’s recommendations (Illumina Inc., San Diego, CA; as previously described [1]) with the following exceptions: 1) 500 ng of cDNA was sheared using a Covaris S220 DNA Sonicator (Covaris, INC. Woburn, MA) to a size range between 200 and 400 bp. 2) Four PCR reactions were amplified to enrich for proper adaptor ligated fragments and properly index the libraries. 3) The final size selection of the library was achieved by an AMPure paramagnetic bead (Agencourt, Beckman Coulter Genomics #A63882, Beverly, MA) cleanup targeting 300-500 bp. The concentration of the library was accurately determined through qPCR according to the manufacturer’s protocol (Kapa Biosystems, Inc., Woburn, MA) to produce a cluster count appropriate for the Illumina platform. The library was indexed and loaded along with 5 other libraries into two lanes of a HiSeq2000 version 3 flow cell. 2 X 101 bp read pairs (later clipped to 100 bp using Consensus Assessment of Sequence and Variation [CASAVA, version 1.8]) were generated for the sample, producing 84,215,192 reads. 16-day adult males were used because based on available RNA-Seq datasets, they provide, on average, the most expressed genes with the highest coverage.

Analytical processing of the Illumina 100 bp reads began by using DUST to filter out regions of low compositional complexity and to convert them into Ns [51]. An in-house Perl script was used to remove Ns, which discards reads without at least 60 bases on non-N sequence. Sequences from host (pig genome; Sscrofa9.2, GCA_000003025.2 from GenBank [52]), bacteria (GBBCT from GenBank [52]), were screened. The resulting 42,107,596 cleaned RNA-Seq reads were mapped to the assembled *A. ceylanicum* genome using Tophat [53] (version 1.3.1), and calculating depth and breadth of coverage using Refcov (version 0.3, http://gmt.genome.wustl.edu/packages/refcov/install.html). The number of reads associated with each gene was determined using HTSeq-Count [54], for each of the annotations. The same mapping and counting process was used to quantify mapping rates to the previously published *A. ceylanicum* genome [31].

The StringTie de novo transcriptome assembly tool [55] was used to assemble the same Illumina RNA-Seq reads into 14,611 StringTie contigs (assembled Illumina transcripts), which were then aligned to the *A. ceylanicum* genome assembly using HISAT [56]. The overlap of both individual mapped Illumina reads and assembled Illumina transcripts with the annotated predicted gene sets was calculated with custom PERL scripts.

## Additional files

Additional file 1: Figure demonstrating the differences in UTR lengths between AC-Orig and AC-PB for (A) 3’ UTRs and (B) 5’ UTRs. (TIFF 493 kb)

Additional file 2: Table of KEGG annotations for AC-Orig and AC-PB. (XLSX 1706 kb)

## Abbreviations

CCS: Circular consensus sequence
PacBio: Pacific Biosciences
SMRT: Single-molecule real-time
UTR: Untranslated region
ZMWs: Zero-mode waveguides
