# Supplementary figures and images for: Improving eukaryotic genome annotation using single molecule mRNA sequencing
Source: BMC Genomics. 2018 Mar 1;19:172. doi: 10.1186/s12864-018-4555-7 (PMC5833154; doi:10.1186/s12864-018-4555-7)

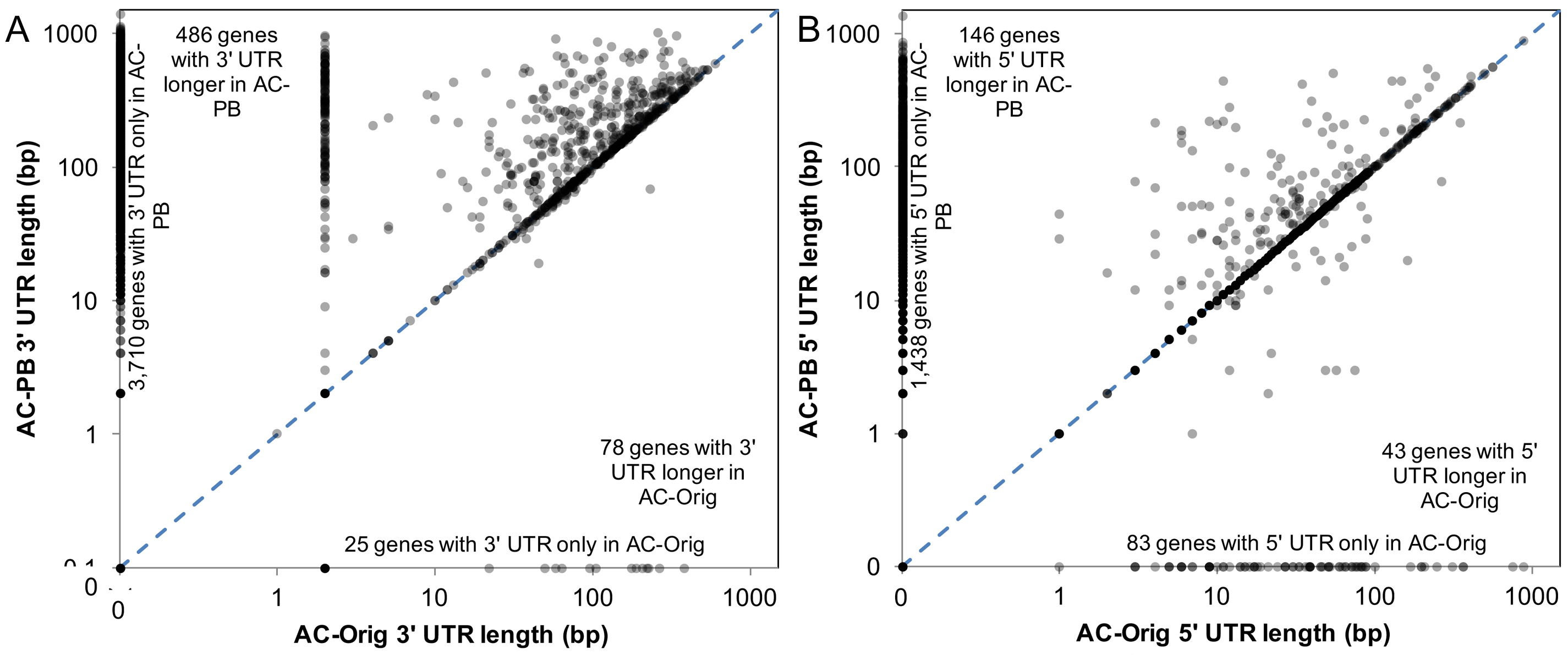

Supplement: Supplementary file 1 — Figure demonstrating the differences in UTR lengths between AC-Orig and AC-PB for (A) 3’ UTRs and (B) 5’ UTRs. (TIFF 493 kb) [file 12864_2018_4555_MOESM1_ESM.tif]
